# Psychometric properties of eysenck personality questionnaire-revised (EPQ-R) short scale in Arabic among undergraduates in Kuwait

**DOI:** 10.1192/j.eurpsy.2021.1990

**Published:** 2021-08-13

**Authors:** B. Alansari, T. Alali

**Affiliations:** Psychology, Kuwait University, Shuwaikh, Kuwait

**Keywords:** EPQR-S, Kuwait University undergraduates, psychometric properties

## Abstract

**Introduction:**

The 48-item EPQR-S is a short version of EPQ-R widely used to assess neuroticism (N), extraversion (E), psychoticism (P) and Lie scale (L) for research purposes. The EPQR-S was chosen for the Arab population because it is a well-established Eysenck theory of personality.

**Objectives:**

To evaluate the psychometric properties of the Arabic EPQR-S.

**Methods:**

The EPQ-R S, the Eysenck Personality Questionnaire (EPQ) and NEO Five-Factor Inventory (NEO–FFI-3) were administered to 1842 (538 males, 1304 females) Kuwait University undergraduates with a mean age = 20.42 ± 1.42. The internal consistency reliability, factor structure, and convergent validity of the EPQR-S with EPQ and NEO–FFI-3 were assessed.

**Results:**

Cronbach’s alpha was satisfactory for N (0.76), E (0.72), L (0.70) and low for P. (0.60). The results revealed significant gender differences in P & E with a favor for males and in N & L a favor with females. PCA showed that EPQR-S four factors explains 52.48% of the total variance. Moreover, the high correlations between the EPQR-S and EPQ scales, with coefficients of (0.92) for the N, (0.88) for the E, (0.78) for the L, and (0.76) for the P as the majority of items of the dimensions of the EPQR-S are the same with those of the EPQ. Furthermore, there were high correlations between the same scales of the EPQR-S and NEO–FFI-3, with coefficients of (0.67) for the N scales, and (0.52) for the E scales.

**Conclusions:**

The findings support the psychometric properties N, E, L scales only.

**Disclosure:**

No significant relationships.

